# Observational study evaluating the association of hoof trimming with dairy cattle behavior and milk yield in Canada and the United Kingdom

**DOI:** 10.3168/jdsc.2023-0493

**Published:** 2024-02-01

**Authors:** G. Stoddard, G. Cramer

**Affiliations:** 1 Department of Veterinary Population Medicine, University of Minnesota, Saint Paul, MN 55108; 2 Purina Animal Nutrition, Arden Hills, MN 55126

## Abstract

The objective of this observational study was to describe the associations between preventive hoof trimming (HT) of cows with no lesions and several behavior- and productivity-outcome measures including activity, lying behavior, and milk yield. A convenience sample of 4 freestall farms in the United Kingdom (n = 2) and Canada (n = 2) were recruited for the study. Inclusion criteria for herds included having a regular trim schedule and use of a specific pedometer system. Cows were trimmed as per their farm's regular protocol and schedule and all HT events were recorded. Only cows with no lesions present on the first recorded HT event were used in the analysis. Activity (steps/d), resting time (min/d), and resting bouts (bouts/d) were recorded daily by pedometers and total daily milk yield (kg/d) was recorded by the farm's milk recording system. Cow-level data such as DIM, breed, and parity were collected from the farm's herd management software. A generalized linear mixed repeated measures model with an exchangeable correlation structure was built to compare the outcome for 7 d following HT to baseline measures for each outcome of interest. For each outcome the average from the 5 d before HT was used as a baseline. All models included the baseline status, lactation number, season, farm, and DIM as covariates. Days from HT and its interaction with farm were included in all models. A total of 1,573 cows with no lesion recorded in their first HT were included in the analysis. Activity and resting time were inversely related on the day of HT, with activity increasing on 3 out of 4 farms (+59 to +84 steps/d) and resting time decreasing (−28 to −52 min/d). Following the day of HT, activity decreased to levels lower than those reported at baseline and resting time increased compared with baseline on most farms. There was a reduction in milk yield on the day of HT on 3 out of 4 farms and a reduction in average yield across the 7 d following trimming for all farms (range: 0.6 to 1.3 kg/d). Results were found to be farm dependent. The impact of the degree of change from baseline in behaviors and milk yield reported in this study on welfare of the cow is unclear, but the results suggest that alterations in the outcomes evaluated can persist beyond the day of trimming compared with baseline, necessitating further investigation.

## Highlights

• Associations between HT and resting and activity behaviors and milk yield were farm dependent.
• Numeric increases in activity and decreases in lying behaviors occurred on the HT day and generally returned to baseline values the day after trimming.
• The magnitude and direction of milk yield change on the day of HT differed across farms.
• Milk yield was reduced for between 1 and 7 days post-HT compared with baseline values.

## Graphical Abstract

**Figure:**
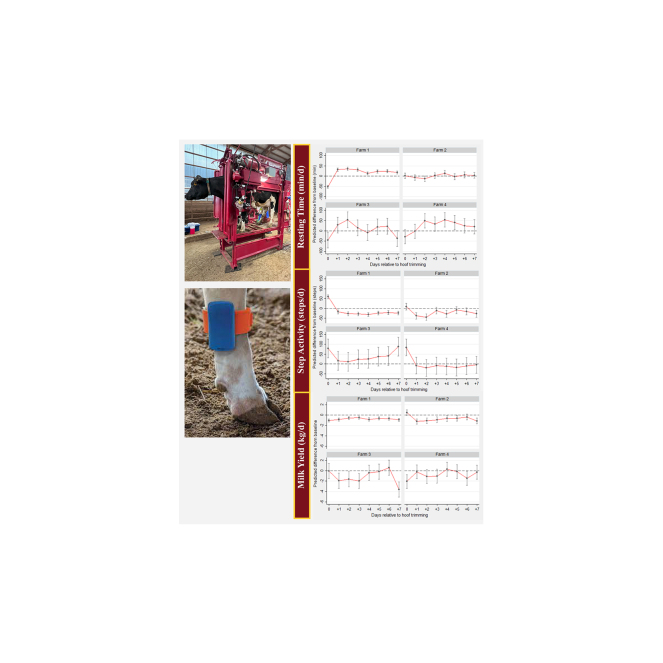
**Summary:** The study describes associations between preventive hoof trimming (HT) of nonlesion dairy cows and resting time, step activity, and milk yield on 4 commercial herds. Differences between the baseline values (5-day average before HT) for all outcomes and values on the day of HT and the 7 days following HT were analyzed. Resting time and activity had an inverse relationship, with resting time decreasing and activity increasing on the day of HT compared with baseline. Average resting time across the 7-day post-HT was greater on most farms than baseline. Milk yield changes on the day of HT were highly variable by farm, but all farms had an average decrease in yield (0.6-1.3 kg/d) across the 7 days post-HT. Results were farm dependent, possibly due to differences in the HT process and time away from pen, requiring further study.

Lameness is considered one of the leading welfare concerns in the dairy industry (von Keyserlingk et al., 2009). It is a costly ($100–$220/cow; Cha et al., 2010; Liang et al., 2017), painful (Whay et al., 1998), and highly prevalent, with prevalence estimated between 20% and 55% in North America (Espejo et al., 2006; von Keyserlingk et al., 2012; Solano et al., 2015) and 7.3% to 60.6% in the UK (Randall et al., 2019). The high cost of lameness is due to multiple factors, including milk production loss (Warnick et al., 2001; Green et al., 2002; Hernandez et al., 2005), decreased reproductive performance (Bicalho et al., 2007; Peake et al., 2011; Hudson et al., 2014), increased culling (Booth et al., 2004; Bicalho et al., 2007), and veterinary treatment and labor costs (Liang et al., 2017). Moreover, lameness can alter a cow's natural behavioral expression, caused in part by a pain response (Whay et al., 1998; Tadich et al., 2013; Bustamante et al., 2015). Lameness causes a reduction in activity and walking speed along with an increase in resting time and in locomotion score (Chapinal et al., 2009; Gomez and Cook, 2010; Navarro et al., 2013). These behavioral changes indicate a reduced state of animal welfare (Dawkins, 2003) and further highlight the need for the implementation of preventive practices.

A recommended preventive practice for lameness is hoof trimming (**HT**) (Shearer and van Amstel, 2001). It is estimated that 85% of US herds have cows trimmed at least once a year (NAHMS, 2007). In the UK it is estimated that 82% of farms do preventive trimming (Pedersen et al., 2022). Despite how commonplace HT is on commercial dairies, few scientific research studies have investigated the relationship between HT and dairy cattle welfare (Stoddard and Cramer, 2017). Physiological data have shown that HT causes an acute stress response (Rizk et al., 2012; Korkmaz et al., 2014), indicated by a rise in respiratory and heart rates, as well as increases in plasma cortisol, lactate, and glucose concentrations. It is possible that this acute stress response induces changes in the behavior of the cow following HT. Previous behavioral studies have shown increases in resting time, decreases in activity, and increases in locomotion score (Chapinal et al., 2010a,b; Van Hertem et al., 2014) following HT, as compared with before HT. This would suggest that the HT process could be stressful and painful and has a negative effect on the well-being of the animal, and the cow is showing compensatory behaviors following HT. However, these previous studies included lame animals, which likely influenced the observed changes, and it is unclear if the same behavioral changes would occur after HT in nonlame animals. For HT to be appropriate as a preventive practice for a nonlame cow, the behaviors associated with lameness should not be present immediately following the procedure, thus investigation into the impact of HT on behaviors of nonlame cows is warranted.

The objective of this study was to describe the associations between preventive HT of cows with no lesions and some behavioral- and production-related outcome measures, including activity, resting behaviors, and milk yield in commercial dairy herds.

The procedures in this observational study were approved by the University of Minnesota Institutional Animal Care and Use Committee (1411–32094A). The study used a convenience sample of 4 Holstein herds using the AFI-PLUS or AFI-ACT2 pedometer system (Afimilk, Afikim, Israel) located in the United Kingdom (n = 2) and Ontario, Canada (n = 2). The pedometer system used allowed for the collection of activity and resting time data (Borchers et al., 2016). Participating herds were required to have a regular HT schedule, with herds routinely trimming cows either at dry-off and as needed (n = 2) or at mid-lactation and dry-off (n = 2) during lactation. Two of the farms used a mattress surface bedded with sawdust, one used deep bedded sand bedding, and one used a combination of sand and sawdust bedding on a mattress surface. The herd sizes ranged from 104 to 1,150 cows. The average 305-d milk production for the herds ranged from 11,137 to 13,691 kg.

Cows were hoof trimmed according to the farm's regular schedule and technique with no intervention from the research team. Hoof trimming techniques on all farms were variations of the functional HT technique (Toussaint-Raven, 1989).

All the farm's HT data, describing the presence or absence of lesions, were collected from April 2015 to July 2016. Only the first nonlesion HT of a cow (i.e., a routine or as-needed trim during the lactation or a trim a dry-off) was included in the analysis. Additional HT events or data from HT that had lesions were not included in the analysis. Other cow-level data such as DIM, breed, and parity were obtained from the farm's recording herd management software (DairyComp 305; Valley Agricultural Software, Tulare, CA). Activity (steps/d), resting time (min/d), and resting bouts (bouts/d) were recorded daily by either AFI-ACT pedometer PLUS tags or AFI-ACTII tags (AfiMilk, S.A.E. AFIKIM, Kibbutz Afikim, Israel). In addition, milk yield (kg) data were collected at each milking using automatic milk meters (AFIFarm, Afimilk, S. A. E. AFIKIM) and the total daily milk yield (kd/d) was calculated. All data were captured automatically every time the animal entered the parlor for milking. These behavioral and milk production data were transferred daily from the AFI herd management software to a text text-based file.

All behavioral, milk yield, cow-level, and HT data management and analyses were performed using STATA 14.1 (Stata Corp., College Station, TX). Daily behavioral and milk yield data were merged with HT and cow-level data. A general linear mixed model was built to compare the outcome for 7 d after HT to the baseline for each of the following outcomes: activity (steps/d), resting bouts (bouts/d), resting time (min/d), and milk yield (kg/d). The baseline represents a 5-d average of the outcome of interest taken from the 5 d before trim event. This 5-d average baseline was included for all outcome models to reduce the impact of the natural variation that occurs in resting time and activity over a lactation (Maselyne et al., 2017). A 7-d follow-up period was used for this study to focus on the acute effects that HT has on the outcomes. The decision to use a 7-d follow-up period was based on graphically evaluating the predictive margins of the null models. On d 7, most behaviors returned to the 5-d baseline or formed a plateau.

For each outcome variable, a generalized mixed model was created that included the following covariates: baseline value of the outcome of interest, lactation number (1, 2+), season (spring [March–May], summer [June–August], fall [September–November], and winter [December–February]), farm ID (1, 2, 3, 4), and DIM. “Days from HT” was forced into all final models. All models included a time by farm interaction. All models included an exchangeable correlation structure to account for the repeated measures on cow, chosen based on the high correlations between different time points. Other correlation structures were evaluated and compared using model AIC (Dohoo et al., 2010). After models were run contrasts of predicted margins were evaluated and graphed by farm to assess the farm by time interaction.

During the 15-mo time period, a total of 7,980 HT events, both lame and trim, were recorded. Of these records, 2,652 (33.2%) were first HT events for a cow (routine and lame trim events). Of the total cows with a first HT events, 1,573 cows did not have a lesion present and had follow-up observations for outcomes of interest and thus were included in the analysis. Behavioral and milk yield data for 88 cows following HT were unavailable as the HT occurred on the day of dry-off and no post-HT milk yield or behavior data were recorded. The cows included in the analysis were, on average, at 171 (95% CI: 166–176) DIM and a lactation of 1.5 (95% CI: 1.4–1.5). Descriptive statistics for the baseline behavioral outcomes (activity, resting time, and resting bouts) and baseline milk yield are presented in Table 1. Lying bout frequency was evaluated, but little variation across the different time points in the study was found, so the data were excluded from further presentation in the article.

**Table 1:** Baseline descriptive statistics for the cows on 4 farms included in the study analyses

Variable^1^	No. of observations	Mean	95% CI
DIM	1,573	171	166–176
Lactation	1,573	1.5	1.4–1.5
Daily milk yield (kg)	1,572	32.5	32.0–33.1
Total activity (steps/d)	1,567	356	350–362
Total resting time (min/d)	1,567	601	594–608
Total resting bouts (bouts/d)	1,567	11	10.8–11.2
305-d mature equivalent milk yield (kg)	1,573	11,482	11,466–11,497

1 Daily milk yield, total activity, total resting time, and total resting bout values are based on the average of the 5-d period preceding hoof trimming.

The mean difference in resting time from baseline to the end of the 7-d follow-up period was dependent on farm (Figure 1). On the day of HT, farm 1 cows had the largest decrease in resting time compared with baseline (−51.8 min/d, 95% CI: −59, −44.1), followed by farm 3 (−44 min/d, 95% CI: −82.7, −6.1) and farm 4 (−28 min/d, 95% CI: −62.6, 5.9). Farm 2 did not see a reduction in resting time. Following HT, resting time increased for cows on most of the farms ranging from 1 to 25 min above baseline.

**Figure 1:**
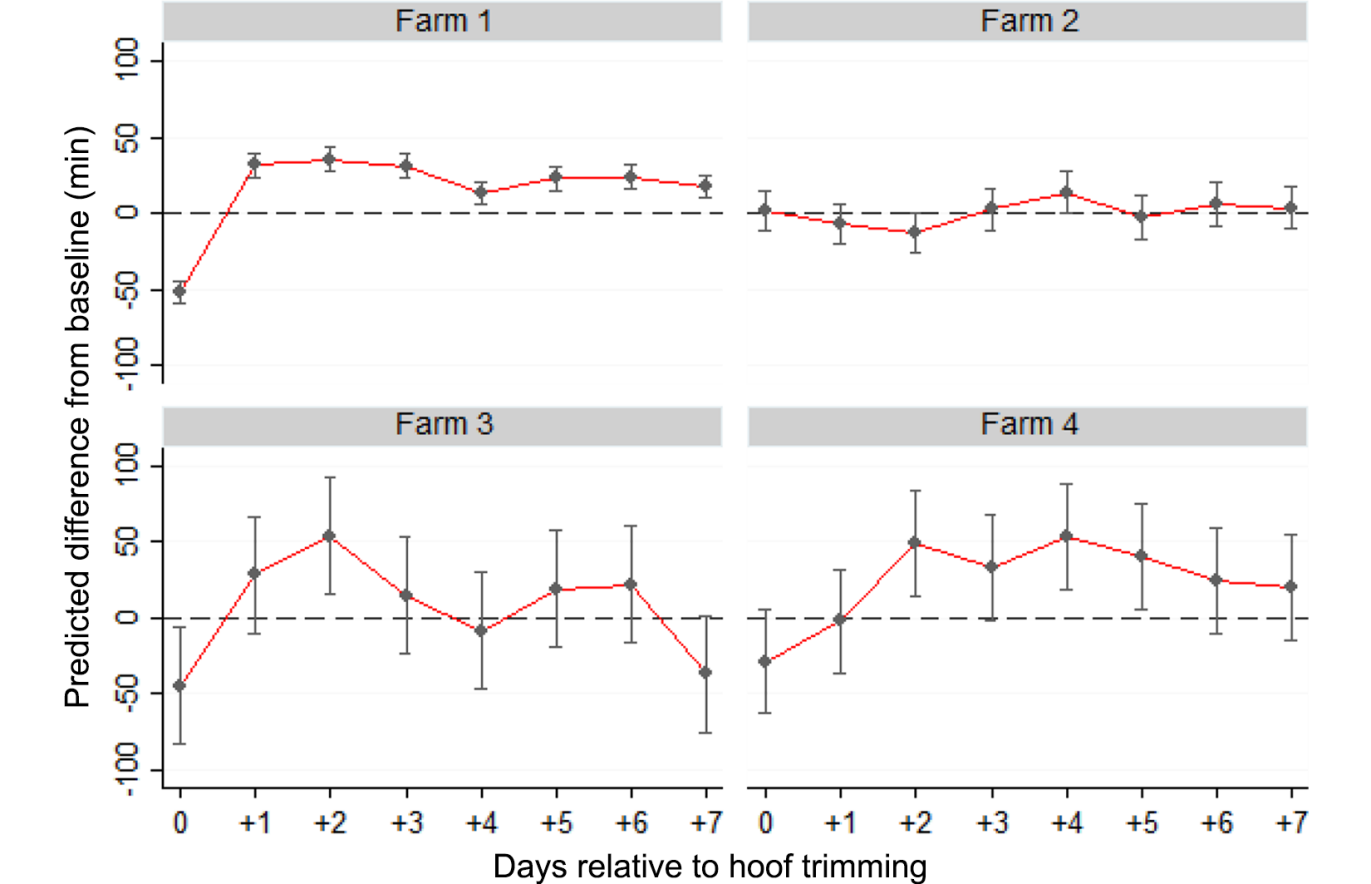
Predicted differences (estimate, 95% CI) for total resting time (min/d), by farm, for the day of hoof trimming (d 0) and the 7 d following hoof trimming compared with baseline. Baseline values were calculated as the average resting time across a 5-d period before hoof trimming.

Activity had an inverse relationship to resting time, with an increase in step activity on the day of HT compared with baseline. For all farms, activity increased on the day of HT, ranging from +9 steps/d (95% CI: −7.4, 25.2) in farm 2 to +84 steps/d (95% CI: 41.4–126.3) in farm 4. All farms returned to levels that were similar to or lower than baseline with the exception of farm 3, which remained above baseline for the 7 d following HT. The average change in activity across the 7 d following HT was dependent on farm and ranged from −25 to 35 steps relative to baseline levels.

The impact of HT on milk yield was dependent on farm. Figure 2 shows the difference in estimated adjusted mean milk yields from the baseline period by farm. On the day of HT cows showed a change in milk production, but it varied in magnitude and direction among the 4 farms. Farms 1 and 4 had a decrease in milk yield on the day of HT of −1.03 kg (95% CI: −1.33, −0.73) and −2.05 kg (95% CI: −3.11, −0.73), respectively. Farm 2, by contrast, had a 0.49 kg (95% CI: −0.02, 0.99) increase in milk yield on the day of HT. Farm 3 had no evidence of a change on the day of HT, but had the largest average reduction across the 7 d following HT. The average milk yield across the 7 d following HT was lower on all farms than at baseline at between −1.3 kg/d (farm 3) and −0.6 kg/d (farm 4).

**Figure 2:**
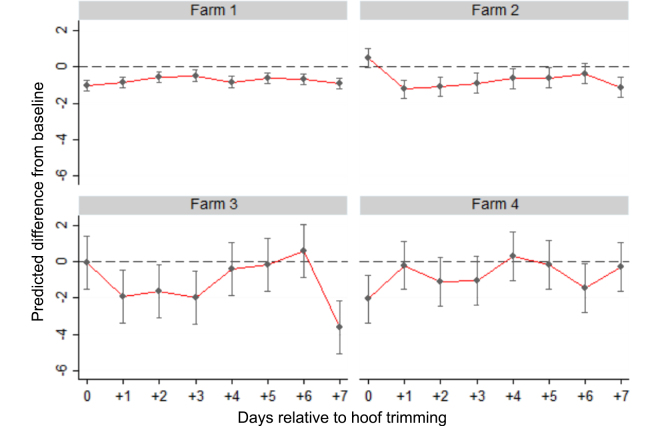
Predicted differences (estimate, 95% CI) for milk yield (kg/d), by farm, for the day of hoof trimming (d 0) and the 7 d following hoof trimming compared with baseline. Baseline values were calculated as the average milk yield across a 5-d period before hoof trimming.

Our results indicate that changes in the cows' lying time and activity behavior were associated with preventive HT, but the magnitude and direction of these associations was dependent on the farm. These results indicate that on certain farms, HT may have a negative impact on the welfare of the animal.

On the day of HT, an increase in activity and a corresponding decrease in resting time on all farms was the only consistent association with HT in our study. This is an expected observation due to the change in a cow's daily routine that HT creates. This increase in activity on the day HT has also been shown in previous research (Chapinal et al., 2010b; Van Hertem et al., 2014). The farm level variation is likely due to differences in how long cows are away from their regular pens.

Similar to previous research (Chapinal et al., 2010a,b; Van Hertem et al., 2014), cows on 3 farms had a decrease in activity on the days following HT. We hypothesize a decrease in activity on the day following HT occurs because the cow compensating for an increase in activity on the day of HT. However, based on the association of a decrease in steps for at least 7 d on 3 of the 4 farms following HT, we hypothesize that events associated with the HT process caused this decrease; however, the exact reason as to why these cows would have this decrease in activity following HT is unclear. Possible reasons that HT is causing a change in behavior for a longer time period include the cow is acclimating to changed weight bearing between the hooves or HT creates pain or inflammatory response, or the decreased resting time on the day of HT. It is important to note, however, that it is unclear what the biological impact of a decrease (5–44 steps/d) or, in the case of the fourth farm, an increase (12–89 steps/d) in daily step activity has on the welfare of the cow. This change in activity appears to be farm dependent. Future work is warranted to determine what aspects of the HT process may be causing this behavior change to find ways to mitigate this change in behavior and potentially improve the welfare of the cow following HT.

On the day of HT, the association between milk yield and HT was variable in magnitude and direction, depending on the farm. On 2 farms an important decrease in milk production was observed. These results differ from the study by Van Hertem et al. (2014) where no evidence of a difference in milk yield was observed. We hypothesize this association was compensatory due to stress during the HT process or by a decreased feed intake and resting time due to less time in the home pen. More controlled studies are required to confirm or refute this hypothesis. Interestingly, the average daily yield across the 7 d following HT remained below baseline values for all 4 farms. This may point to longer term impacts on milk yield following periods of restricted access to lying and feed, particularly when time away from the home pen is longer; however, time away from the pen was not measured in the current study. These reductions may also reflect the long-term impact of stress from certain routine management practices such as HT that warrant further investigation.

Possible explanations for the difference between farms for all of our outcomes post-HT include differences in HT technique and hoof trimmer experience level. Additionally, since the farms all had different management and environmental factors, it is likely that walking distances, time away from stalls, and feed were different.

Our study did have some limitations due to study design. Since this study was observational in nature, we must be cautious ascribing causality. In addition, since a specific housing system was chosen (freestall barns), it limits the generalizability of these results to other housing systems. Cows were also not locomotion scored before entering the study. It could be possible that lame cows not displaying a hoof lesion entered the study; however, we expect this to apply to a small proportion of cows since lesions are the most common cause of lameness (Leach et al., 2012; Green et al., 2014). Strengths of the study included the use of multiple farms located in 2 different regions of the world with different hoof trimmers on each farm and the duration of the study, allowing for more generalizable results. This strength offsets the limitation that environmental or management changes could explain the changes from baseline.

This study showed that preventive HT of cows without lesions was associated with short-term changes in cows' resting time and activity behavior and milk production, but the direction and magnitude of these changes were dependent on the farm. The impact of these behavioral changes and milk production on the welfare status of the cow is not completely clear. To minimize the impact of HT on the cow, various HT techniques and approaches to handling and restraint should be investigated to determine what effect they may have on behavior and milk production, and to determine what is most efficacious for the welfare of the cow.
